# Observations on the associations between damaging and aggressive behaviors, related lesions, and their implications for the welfare of pigs in the grower-finisher period

**DOI:** 10.3389/fvets.2025.1523663

**Published:** 2025-03-24

**Authors:** Lucy Markland, Julia Adriana Calderón Díaz, Laura Ann Boyle, Joana Pessoa, Nienke van Staaveren

**Affiliations:** ^1^Pig and Poultry Research and Knowledge Transfer Department, Teagasc Animal and Grassland Research and Innovation Centre, Fermoy, Ireland; ^2^School of Veterinary Medicine, University College Dublin, Dublin, Ireland; ^3^National Food Institute, Technical University of Denmark, Kongens Lyngby, Denmark; ^4^Department of Population Health Sciences, Faculty of Veterinary Medicine, Utrecht University, Utrecht, Netherlands; ^5^Department of Animal Biosciences, Ontario Agricultural College, University of Guelph, Guelph, ON, Canada

**Keywords:** fighting, injury, early warning signals, swine, welfare, tail ear and flank biting

## Abstract

**Introduction:**

Pigs perform damaging and aggressive behaviors, but few studies investigated associations between behaviors and resulting lesions in intensive settings. We investigated such associations within and across production stages to understand implications for welfare, and interpreted cut-off values of behavior for use as warning signals.

**Methods:**

Four batches of 419 pigs each (*n =* 1,676 pigs) were followed on arrival to a commercial grower-finisher unit at 12 weeks of age until slaughter. Pigs had docked tails, were managed according to routine practice and housed in 48 mixed-sex groups in eight rooms [35(±2) pigs/pen; 6 pens/room/batch]. Ear and tail lesions were assessed when pigs arrived to grower stage I [24.9 ± 5.33 kg of body weight (BW)], after 2 weeks when transferred to grower stage II (33.3 ± 7.04 kg BW), and after 4 weeks when transferred to the finisher stage (60.2 ± 7.74 kg BW; 18 weeks of age). All occurrences of damaging (ear, tail, and flank biting) and aggressive behaviors were recorded for 5 min per pen from the week after pigs arrived for 11 weeks.

**Results:**

High variability existed between pens for behaviors and percentage of pigs that developed new ear or tail lesions on arrival to grower II and finisher stage. There were significant correlations among the behaviors only within grower stage II (all behaviors: 0.65 ≤ r_s_ ≤ 0.80, *p* < 0.05), while the only correlations across production stages were ear biting (grower II and finisher r_s_ = −0.29, *p* < 0.05), flank biting (grower II and finisher r_s_ = 0.70, *p* < 0.05), and aggression (grower I and II r_s_ = 0.37, *p* < 0.05). This suggests a sensitive period during grower stage II but also that performance of behaviors changes over time. The frequency of ear and tail biting did not need to be high for new lesions to develop, but thresholds changed depending on stage, behaviors, and lesion type.

**Discussion:**

This underscores the intricacies in developing cut-off values for warning signals and may relate to the cumulative effect of different risk factors. Thus, early identification and multifaceted management strategies tailored to specific pens are needed to address behaviors with adverse implications for pig welfare. This highlights the challenges and complexities of improving pig welfare within current intensive production settings.

## 1 Introduction

Pigs are social animals showing behaviors ranging from positive (e.g., social nosing and play) to negative (e.g., agonistic behavior, oral manipulation) when interacting in a farm setting. The expression of social behaviors is important, for recognition, group cohesion, and hierarchy formation and maintenance (1, 2). These behaviors thus have consequences for pig welfare. Group stress due to aggression or unwanted social interactions is a highly relevant welfare consequence in nearly all categories of pigs in commercial production systems. The same can be said for the inability to express exploratory/foraging behavior, which may be redirected to oral manipulation of other pigs in the pen (3). This is also referred to as damaging behavior and may include tail-, ear-, and flank-directed behavior (4). Both aggressive and damaging behavior can lead to lesions of, respectively, the skin and targeted areas of tails, ears and flanks (4, 5). Soft tissue lesions and integument damage resulting from these behaviors are a highly relevant welfare consequence (3).

Pig producers consider it important to manage damaging behaviors and the associated lesions (6–9), perhaps more so than aggressive behavior which appears to be somewhat more accepted or considered sufficiently controllable (7, 8). Historically, tail biting received the most attention of all damaging behaviors, but in recent years there is growing research attention on ear lesions (4, 10, 11). In light of the ban on routine tail docking, tail biting in particular received much research attention to identify risk factors and ways of managing this multifactorial problem (12–14). Consequently, more is known about the economic implications of the presence of tail lesions on farm profitability (15, 16) and costs of implementing potential management measures (17, 18), which may further (de)motivate producers to address this issue. Ear lesions have a more complex etiology as unlike tail lesions, overt ear biting is not an immediate cause; pathogens associated with ear necrosis are also implicated (4, 11, 19). Similarly, lesions on the flanks of pigs may be due to sustained “nosing” followed by infection as opposed to overt “biting” (20, 21). Interestingly, the link between damaging behaviors and health status is also receiving more attention [reviewed by Boyle et al. (4)]. Despite the aforementioned research, damaging and aggressive behaviors are still highly prevalent on pig farms (21–24).

To address these issues, it is necessary to recognize and monitor either the behavior and/or the associated lesions or “animal-based measurements” (3). Bracke et al. (6) reported that Dutch pig farmers considered the level of tail biting as severe when they observed one animal showing a tail wound of any severity. However, the presence of a lesion indicates that the problem is already present. Furthermore, the likelihood of farmers entering pens to perform detailed pig tail assessments for earlier detection is unlikely (14). Earlier intervention based on behavioral observations may be more appropriate, but is generally considered difficult due to the sporadic nature of tail biting (9) and the limited resources (labor, time etc.) available to conduct such detailed observations in commercial production systems. Alternatively, with the expansion of precision livestock farming there are efforts underway to develop automated detection of aggressive or damaging behaviors [reviewed by Matthews et al. (25), Gómez et al. (26), and Siegford (27)]. However, these technologies are so far only validated to a limited extent, are not yet commercially available and may not be practical or economically feasible to install on commercial pig farms (18, 26, 27).

Disturbances and restlessness in pens where damaging behavior occurs, may also give rise to aggressive interactions. For example, providing pigs with opportunities to express exploratory behavior was associated with less tail biting and less tail lesions, but also less aggressive behavior (28, 29). Ear biting is more likely to provoke an aggressive response (30). Tail and ear biting are linked, potentially due to shared risk factors [e.g., lack of enrichment or poor health status (10)], and tail and ear lesions can co-occur (24). Both Beattie et al. (31) and Brunberg et al. (32) found that pigs that performed tail biting also performed more ear biting, and to some extent also more belly nosing. Telkänranta et al. (33) reported positive, albeit low, correlations between prevalence of tail and ear lesions at individual and pen-level. Depending on the time period assessed and the environment, Ursinus et al. (34) also found correlations between tail biting/tail damage in a pen and levels of tail biting, fighting and ear biting in other time periods. Additionally, pigs with tail lesions at weaning were more likely to remain victims of tail biting in successive stages (up to 21 weeks of age) when housed in barren pens (34). Based on survey data on tail and ear biting outbreaks, farmers report similar correlations between tail and ear biting both within and, at times, across production stages (9). Therefore, the aim of this study was to investigate how behaviors and lesions are associated within and across production stages to better understand implications for pig welfare and their potential as practical “early-warning” support tools.

## 2 Methods

### 2.1 Ethics statement

The study had ethical approval from the Teagasc Animal Ethics Committee (TAEC 204/2018).

### 2.2 Experimental design and animal husbandry

This was an observational study, whereby pigs were managed as per routine farm practice. The study was conducted from July to November 2018. A total of 1,676 (*n =* 773 females and *n =* 903 males) 12-week old Large White × Landrace weaner pigs [24.9 ± 5.33 kg of body weight (BW)] with docked tails were individually ear tagged upon arrival to the farm. Pigs were housed in eight rooms each divided into six pens, forming a total of 48 mixed sex groups [35 (±2) pigs per pen] and followed until slaughter at 114.9 ± 11.79 kg of BW. The study was conducted using four batches of approximately 419 pigs each that arrived to the farm over a 6 week period. Pigs originated from a commercial farrow-to-wean farm in Ireland and were transported to a separate grower-finisher unit at 12 weeks of age. On arrival, pigs were mixed and moved to the first grower stage where they remained for 2 weeks. Pigs were then moved to the second grower stage (33.3 ± 7.04 kg BW; 14 weeks of age), and after 4 weeks were transferred to the finisher stage accommodation (60.2 ± 7.74 kg BW; 18 weeks of age) where they remained until slaughter. Once pigs were mixed upon arrival to the farm, they were kept in the same groups throughout the production stages (i.e., they were not remixed between stages). The farm was positive for *Mycoplasma hyopneumoniae* (Mhyo), *Actinobacillus pleuropneumoniae* (APP), porcine reproductive and respiratory syndrome virus (PRRSv) and Influenza A virus (IAv) and vaccinated for Mhyo, PRRSv, and IAv. Pens in all production stages had fully slatted concrete floors whereby feed was delivered regularly to a wet-feed trough where approximately seven pigs could feed simultaneously (Hydromix wet feeding system, Big Dutchman, IDS, Portlaoise, Co. Laois, Ireland—ratio five pigs to one feeder space). The level of feed in the trough was monitored continuously via a probe such that feed was topped up whenever the level fell below a certain limit thereby ensuring constant availability of feed. Water was available *ad libitum* via two nipple drinkers. Space allowance was compliant with EU legislation throughout all production stages, providing between 0.55 and 0.76 m^2^ per pig. All rooms were artificially illuminated from 0800 to 1700 h. Environmental enrichment was present in each pen as provided by the farm staff in the form of hard plastic balls and chains (one of each per pen) suspended from the pen partitions, hanging at an accessible height for the pigs. All pens received the same type and amount of enrichment so there was no variation between pens. Enrichment objects remained the same for the duration of the study.

### 2.3 Measurements

#### 2.3.1 Behavioral observations

Each pen (*n =* 48) was observed directly for 5 min weekly for 11 weeks starting the week after the pigs arrived at the farm. The observation schedule was designed so that one person could observe all 48 pens for 5 min (i.e., 4 h per day). At each behavior assessment, the observer would first enter the room and ensure all pigs were awake, and would wait 5 min to start the observation. The observation method chosen was informed by previous work (35, 36) and practical constraints. Observations were made between 0900 and 1600 h on the same day each week with the order of observation randomized each week to ensure that every pen was balanced across each of the 7 h available. The observer carrying out the behavior observations was trained by author LAB who has 30 years of experience in pig behavior research. Due to turnover in research staff, a second observer was trained in a similar manner and responsible for the observations at week 5 and onwards. Inter-observer reliability between LAB and each observer was >0.80. Within each pen, all occurrences of tail-, ear-, and flank-directed behavior as well as aggressive interactions were counted (hereafter referred to as tail, ear, flank biting and aggression). An ethogram for all recorded behaviors is presented in Table 1.

**Table 1:** Ethogram of behaviors recorded during 5-min per pen continuous observations during the grower-finisher period on 48 mixed sex groups (*n =* 1,676) of pigs on a commercial farm.

Behavior	Definition
Tail-directed (“tail biting”)	Tail in the mouth of another pig: ranges from tail being gently manipulated to tail being chewed/bitten
Ear-directed (“ear biting”)	Ear in the mouth of another pig: ranges from ear being gently manipulated to being chewed/bitten
Flank-directed (“flank biting”)	Oral/nasal attention including bites and nosing, directed toward the flank of another pig
Aggressive interactions (“aggression”)	Mutual pushing parallel or perpendicular, ramming or pushing of the opponent with the head, with or without biting in rapid succession.

#### 2.3.2 Lesions

Each individual pig was inspected by author JP for ear and tail lesions on arrival at the farm, and on transfer to the second grower and finisher stages. At these times, all pigs were weighed and could be individually assessed for lesions in good visibility without increased handling of the pigs or interrupting the commercial practices of the farm. *Ear lesions* were scored using a modified version of the 5-point scoring system described by Diana et al. (35) where 0 = no lesion; 1 = mild lesions (superficial bites but no blood); 2 = moderate lesions (evidence of bites/teeth marks with fresh blood) and/or infection; 3 = severe (partial total loss of the ear); and 4 = very severe (total loss of the ear). *Tail lesions* were scored as per Harley et al. (37) on a 5-point scale where 0 = no evidence of tail biting; 1 = evidence of chewing or puncture wounds, but no evidence of swelling; 2 = evidence of chewing with swelling and signs of possible infection; 3 = partial loss of the tail and 4 = total loss of the tail.

### 2.4 Statistical analysis

Pen was considered the experimental unit (*n =* 48). All data analyses were conducted in R v4.4.1 (38).

We previously reported in detail on the prevalence of tail and ear lesions (24). Tail and ear lesions assessed for each individual pig were reclassified as score 0 and score ≥ 1 due to the low number of observations of higher scores. To assess associations between behavior and lesions, we were in particular interested in newly developed lesions at each stage. For each pen, the proportion of pigs with new tail and ear lesions on transfer to the second grower and finisher stages was calculated (i.e., pigs with score 0 that upon the next transfer had a score ≥ 1 for each respective lesion type).

For each production stage, the frequencies of each behavior assessed overtime at pen-level were averaged. Meaning that for the first grower stage the average of two behavior assessments were considered, while for second grower, the average of four assessments were considered, and for finisher stage five assessments.

Spearman’s rank correlation coefficients (r_s_) were employed to examine potential associations among the four different behaviors both within and across subsequent production stages. Correlations with |r_s_| ≤ 0.3 were classified as low, those with 0.3 < |r_s_| ≤ 0.5 were considered moderate, and correlations with |r_s_| > 0.5 were categorized as strong. Regression tree analysis was used to identify cut-off values for frequencies of tail and ear biting behaviors associated with the prevalence of new tail and ear lesions using the *rpart* package (39). This would help to identify the primary behavior (and its frequency) that could serve as a warning signal for the development of new lesions. Four separate regression trees were constructed for the proportion of new tail or ear lesions on transfer to the second grower and finisher stages. Each regression tree included the proportion of new tail or ear lesions as outcome variables with the mean frequency of tail and ear biting behaviors performed in the previous stage as the explanatory variables. The stopping criterion was a minimum of 10% of the pens being required to create a branch and/or leaf.

## 3 Results

**Figure 1:**
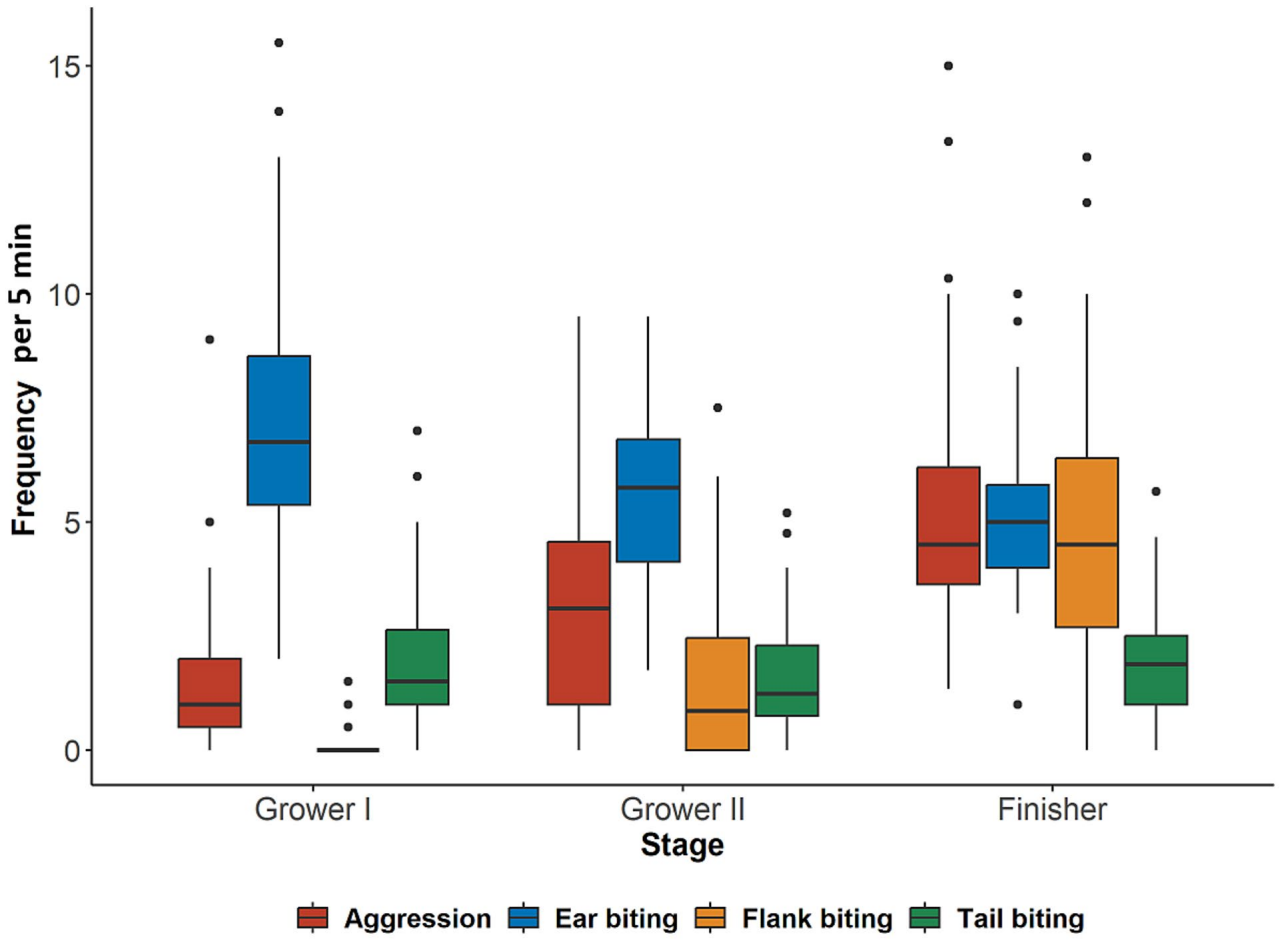
Frequency of behaviors performed by 48 mixed sex groups (*n =* 1,676) of pigs per 5-min observation during the first grower (Grower I, 12–13 weeks of age), second grower (Grower II, 14–17 weeks of age), and finisher (18 weeks of age until slaughter) stages.

Mean frequency for each studied behavior for each production stage is presented in Figure 1 and the frequency of behaviors for each of the 11 weeks is presented in Supplementary Figure 1. In general, mean frequency for all behaviors was low with high variability between pens. Ear biting was the most frequently observed behavior throughout the entire production period. As pigs moved through the production stages, the frequency of ear and tail biting declined with the latter having similar frequencies during the second grower and finisher stages. On the other hand, flank biting and aggressive behavior increased as pigs progressed through the production stages. Within production stages, significant associations (*p* < 0.05) among the behaviors were found only during the second grower stage (Table 2), where all behaviors were highly correlated with each other (r_s_ ≥ 0.65). Additionally, there was a tendency (*p* = 0.0610) for a low positive correlation (r_s_ = 0.27) between flank biting and aggression during the finisher stage. No other associations (*p* > 0.05) were observed among the studied behaviors during the first grower stage or the finisher stage (Table 2).

**Table 2:** Spearman correlations (r_s_) among ear, tail and flank biting and aggressive behavior within production stages (Grower I, 12–13 weeks of age; Grower II, 14–17 weeks of age; Finisher, 18 weeks of age until slaughter) in 48 mixed sex groups (*n =* 1,676 pigs) of grower-finisher pigs in a commercial farm.

Behavior 1	Behavior 2	r_s_	*P*
Grower I			
Ear biting	Tail biting	0.09	0.5446
Ear biting	Flank biting	0.22	0.1317
Ear biting	Aggression	−0.12	0.4354
Tail biting	Flank biting	0.16	0.2899
Tail biting	Aggression	−0.07	0.6532
Flank biting	Aggression	−0.22	0.1334
Grower II			
Ear biting	Tail biting	0.73	<0.0001
Ear biting	Flank biting	0.65	<0.0001
Ear biting	Aggression	0.73	<0.0001
Tail biting	Flank biting	0.72	<0.0001
Tail biting	Aggression	0.72	<0.0001
Flank biting	Aggression	0.80	<0.0001
Finisher			
Ear biting	Tail biting	0.05	0.7597
Ear biting	Flank biting	−0.05	0.735
Ear biting	Aggression	0.15	0.3252
Tail biting	Flank biting	0.16	0.2734
Tail biting	Aggression	−0.02	0.9150
Flank biting	Aggression	0.27	0.0610

Across production stages, there were no associations (*p* > 0.05) between ear, tail or flank biting in the first and second grower stages. Moderate to strong positive correlations were observed between aggression in the first grower stage and ear biting (r_s_ = 0.36), flank biting (r_s_ = 0.54) and aggression (r_s_ = 0.37) in the second grower stage. Additionally, there was a strong positive correlation between aggression in the second grower and flank biting in the finisher period (r_s_ = 0.51), a moderate negative correlation (r_s_ = −0.42) between ear biting in the first grower and tail biting in the finisher stage, as well as a low positive correlation between tail biting and aggression (r_s_ = 0.29) in the same production stages (Table 3). Ear biting during the second grower stage had a low negative association with ear biting and a moderate positive association with flank biting in the finisher stage (*p* < 0.05). Moreover, tail biting, flank biting, and aggression during the second grower stage had strong positive correlations with flank biting during the finisher stage (r_s_ ≥0.54, Table 3).

**Table 3:** Spearman correlations (r_s_) among ear, tail and flank biting and aggressive behavior across production stages (Grower I, 12–13 weeks of age; Grower II, 14–17 weeks of age; Finisher, 18 weeks of age until slaughter) in 48 mixed sex groups (*n =* 1,676 pigs) of grower-finisher pigs in a commercial farm.

Stage/behavior	Grower II				Finisher			
	Ear biting	Tail biting	Flank biting	Aggression	Ear biting	Tail biting	Flank biting	Aggression
Grower I								
Ear biting	0.22	0.14	0.02	0.11	0.05	−0.42^*^	0.06	0.22
Tail biting	0.11	0.22	0.14	0.08	0.14	0.07	0.09	0.29^*^
Flank biting	−0.09	−0.15	−0.20	−0.20	0.23	−0.04	−0.01	0.20
Aggression	0.36^*^	0.27^(*)^	0.54^*^	0.37^*^	−0.16	−0.08	0.51^*^	0.18
Grower II								
Ear biting					−0.29^*^	−0.01	0.48^*^	0.03
Tail biting					−0.18	−0.04	0.54^*^	−0.02
Flank biting					−0.17	−0.03	0.70^*^	0.06
Aggression					−0.22	0.00	0.60^*^	0.06

^*^p < 0.05; (*) 0.05 < *p* < 0.1.

**Figure 2:**
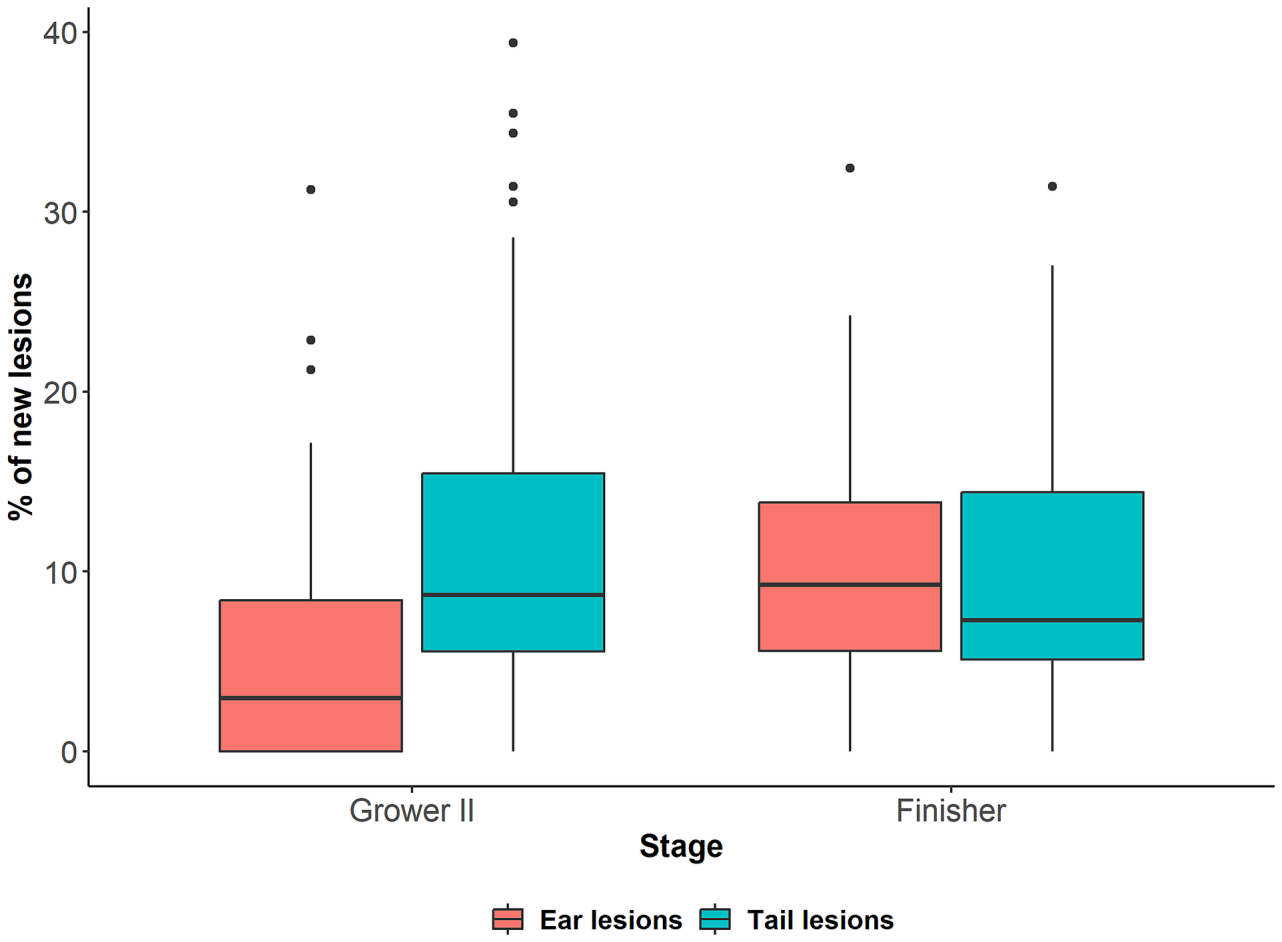
Percentage (%) of pigs with new tail and ear lesions (i.e., score ≥ 1) in 48 mixed sex pens (*n =* 1,676) on arrival to the second grower (Grower II, 14 weeks of age) stage compared to arrival to the previous stage (Grower I, 12 weeks of age), and on arrival to the finisher (18 weeks of age) stage compared to arrival to the previous stage (Grower II).

The total percentage of pigs affected by tail and ear lesions and the pattern of lesion development is presented in van Staaveren et al. (24), but ranged between approximately 28 and 39% for ear lesions and 2.5–12.5% for tail lesions depending on the stage of production. The prevalence of new tail and ear lesions on arrival at the second grower and finisher stages is presented in Figure 2. The mean prevalence of pigs with new tail lesions was similar on arrival to the second grower (11.9%, range: 0–39.4%) and finisher stage (10.1%, range: 0–31.4%). For ear lesions, the mean percentage of pigs with new lesions was higher upon arrival in the finisher stage (10.0%, range: 0–32.4%) than the second grower stage (5.8%, range: 0–31.2%). Regardless of lesion type, large variation in the percentage of pigs with new lesions was observed between pens. The prevalence of new ear and tail lesions for each pen on arrival at the second grower and finisher stages is presented in Supplementary Figures 2, 3, respectively.

**Figure 3:**
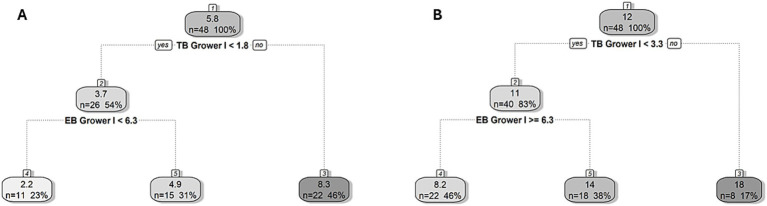
Regression tree for the prevalence of new **(A)** ear and **(B)** tail lesions on arrival to the second grower stage (Grower II, 14 weeks of age). The cut-off value for the frequency of tail (TB) and ear biting (EB) in the first grower (Grower I, 12–13 weeks of age) stage showed the best division of pens in terms of developing new lesions and is indicated in bold underneath the split node. The lefthand split in the decision rule always indicates that the condition was met (“yes”) while the righthand split indicates when the condition is not met (“no”). Within each node/leaf, the top number presents the percentage of pigs with new lesions followed by the number (*n*) and percentage (%) of pens within the group presented underneath for a given decision rule.

Regression tree analysis was used to identify the primary behavior linked to new tail and ear lesions on arrival to the second grower and finisher stages. Cut-off values that resulted in partitions with the greatest differences in lesion prevalence were determined. In both stages, tail biting was the main behavior associated with the occurrence of new lesions. The cut-off values for the development of new tail and ear lesions on transfer to the second grower stage were 3.3 and 1.8 observed instances of tail biting per 5 min in a given pen during the first grower stage, respectively (Figure 3). In pens above the tail biting threshold, the mean prevalence of tail lesions was 18% (as opposed to 12%) while the prevalence of ear lesions was 8.3% (as opposed to 5.8%). Where the frequency of tail biting in a pen was below the threshold, there was a cut-off value for new tail and ear lesions of ≥6.3 and < 6.3 instances of ear biting per 5 min per pen, respectively. In pens below the tail biting threshold, the mean prevalence of tail lesions in pens below the 6.3 ear biting threshold was 14% (as opposed to 11%), while the prevalence of ear lesions in pens above the 6.3 ear biting threshold was 4.9% (as opposed to 3.7%).

**Figure 4:**
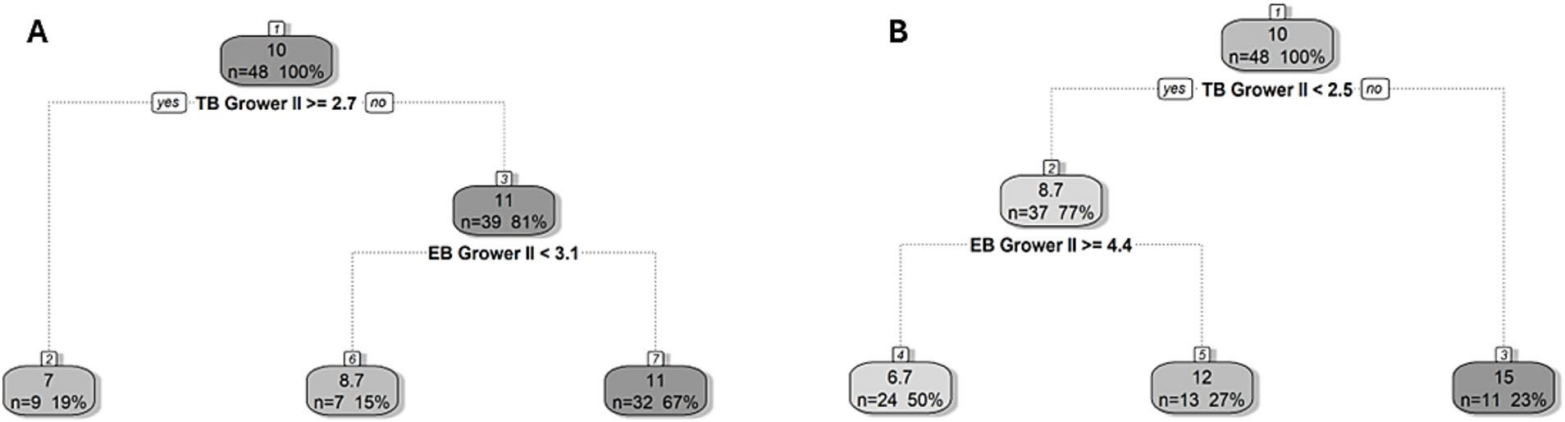
Regression tree for the prevalence of new **(A)** ear and **(B)** tail lesions on arrival to the finisher stage (18 weeks of age). The cut-off value for the frequency of tail (TB) and ear biting (EB) in the second grower (Grower II, 14–17 weeks of age) stage showed the best division of pens in terms of developing new lesions and is indicated in bold underneath the split node. The lefthand split in the decision rule always indicates that the condition was met (“yes”) while the righthand split indicates when the condition is not met (“no”). Within each node/leaf, the top number presents the percentage of pigs with new lesions followed by the number (*n*) and percentage (%) of pens within the group presented underneath for a given decision rule.

Similarly, the cut-off values for the development of new tail and ear lesions on transfer to the finisher stage were < 2.5 and ≥ 2.7 observed instances of tail biting per 5 min in a given pen during the second grower stage, respectively (Figure 4). In pens above the 2.5 tail biting threshold, the mean prevalence of tail lesions was 15% (as opposed to 10%), while in pens below the 2.7 tail biting threshold the prevalence of ear lesions was 11% (as opposed to 10%). Moreover, a cut-off value for new tail and ear lesions of ≥4.4 and <3.1 instances of ear biting per 5 min in pens, respectively was estimated. In the pens below the tail biting threshold, the mean prevalence of tail lesions in pens above the 4.4 ear biting threshold was 6.7% (as opposed to 8.7%), while in pens below the 3.1 ear biting threshold the mean prevalence of ear lesions was 8.7% (as opposed to 11%).

## 4 Discussion

This study examined the associations between behaviors and related lesions in pigs within and across production stages focusing on tail and ear lesions as animal based indicators of major pig welfare problems. The aim was to identify relationships between behaviors, explore the use of behavioral thresholds as welfare indicators as a proof of concept on a single farm, and interpret this in the context of general pig welfare in a commercial setting. These associations can provide insight on the development of abnormal behaviors on a commercial farm, which can help inform management strategies. In addition, knowledge on behavior patterns and cut-off values may be useful in the development of early-warning signals in precision livestock farming technology. Pigs were followed longitudinally on a commercial grower-finisher farm to observe their behavior and assess the prevalence of new tail or ear lesions on transfer between production stages.

Strong positive correlations were found between all behaviors (ear, tail, flank biting and aggression) within the second grower stage (14–18 weeks of age). In contrast, no significant correlations were found within the first grower (12–14 weeks of age) or finisher (18 weeks of age until slaughter) stages. This implies that on this farm these types of behaviors were more likely to co-occur in the second grower stage and may reflect a general vulnerability around this time. It is important to note that this farm had a relatively unique situation in that pigs were transported from the breeder farm at 2 weeks post-weaning and mixed into unfamiliar groups on the farm on which the study was conducted. After the pigs arrived on the farm, a high incidence of respiratory distress was detected during the first grower stage (40). Thereafter the pigs moved relatively quickly (after 2 weeks) into the accommodation associated with the next production stage (second grower) which was considerably different to that used in the previous stage (first grower). Transportation, mixing, exposure to new diseases, and adjusting to multiple new housing types in a short time span are major sources of stress in pigs. It is possible that the pigs were debilitated from the associated stress and disease pressure, while the move to the second grower stage further challenged them, contributing to the co-occurrence of the studied behaviors during the second grower stage. Potentially the shorter time spent in the first grower stage when arriving on the farm could explain the lack of correlations found within this stage but also the fact that the pigs were clinically unwell. The lack of correlations between behaviors in the finisher period, as also observed in van Staaveren et al. (36), may indicate that as pigs age, they settle into a specific behavioral profile or that there are less shared risk factors for the differing behaviors at this stage. It is important to identify problem areas or stages on individual farms to proactively address potential welfare issues.

Few studies looked at correlations between damaging and aggressive behaviors in pigs at various ages. Apart from the aforementioned van Staaveren et al. (36), Beattie et al. (31) previously found low (≤ 0.3) positive correlations between tail biting and both ear biting and belly nosing in pigs up to 7 weeks of age. Others attempted to identify differences in behaviors in pigs classified as “non,” “low,” or “high” performers of tail biting behavior (32, 41). For example, Brunberg et al. (32) found that pigs between 10 and 21 weeks of age that performed more tail biting also showed more ear biting, and to some extent more belly nosing, than pigs who did not perform tail biting. Similarly, Hakansson and Bolhuis (41) observed more ear biting and other biting (excluding ear and tails) in pigs up to 6 weeks of age that were classified as “high biters” based on tail biting behavior compared to “non-biters.” It should be noted, however, that the classifications in the aforementioned studies are often based on relative, arbitrary frequencies of the behavior, and that the number of pigs in the classification groups is unbalanced, particularly with underrepresentation in the “high” performing group (32, 41). This makes comparisons between studies difficult, and warrants caution in the interpretation of results. However, the results from these multiple studies point toward the idea that disturbances and restlessness from damaging behavior may trigger aggressive responses, and the existence of shared risk factors for issues such as tail and ear biting, which may explain the correlations found in the current study (3, 10, 30, 34).

The longitudinal nature of our study allowed us to investigate whether pens showing a high frequency of a certain behavior in the earlier stages would continue to do so in later production stages. Particularly, aggression in the first grower stage was positively correlated with ear biting, flank biting and aggression in the second grower stage, as well as flank biting in the finisher stage. From the behaviors observed in the second grower stage, they were positively correlated with flank biting during the finisher stage.

To the best of our knowledge, only Ursinus et al. (34) investigated the associations between behaviors and tail biting over a pig’s lifetime. They found that being a tail biter in the weaner stage (4–5 weeks of age) did not increase the likelihood of being a tail biter in subsequent phases (grower: 8–11 of age, finisher: 16–21 weeks of age). Similarly, Paoli et al. (42) found little consistency over time in tail-directed behaviors in pigs from week to week (5–8 weeks of age). The lack of correlations in tail biting behavior across stages in our study are consistent with those findings. However, direct comparisons cannot be made, as our behavior observations were not conducted at the individual pig level, making it impossible to assess whether the same pig(s) performed the damaging or aggressive behaviors over time as done in these studies (32, 34, 41). Still, we reason that the similar findings across studies indicate that “tail-biter pigs” and “tail-biter pens” may not be a common occurrence, pointing to the need to consistently monitor all pens overtime to identify potential tail biting outbreaks at an early stage.

Interestingly, Ursinus et al. (34) observed a negative correlation (−0.42) between ear biting in the grower stage and tail biting in the finisher stage, which is similar to the negative correlation (−0.42) found between ear biting in the first grower stage (12–14 weeks of age) and tail biting in the finisher stage (18–23 weeks) in the current study. The correlation found in that study was present only when assessing barren pens, while a weaker tendency was reported for enriched pens (34). Still, due to the relatively poor nature of the enrichment items used during our study, the evidence reported by Ursinus et al. (34) seems consistent with our findings. Furthermore, there are suggestions of a trade-off where pigs switch from ear biting in the grower stage to more tail biting in the finisher stage (23). Additionally, the current study found a negative correlation (−0.29) between ear biting in the second grower stage and ear biting in the finisher stage. In contrast, however, Paoli et al. (41) reported that ear biting behavior was highly consistent at group level in pigs 5–8 weeks of age. These pigs remained in the same accommodation throughout, while in our study, pig housing changed between three different production stages. Possibly ear biting could be more sensitive to external factors than the result of mainly individual differences. The pigs in the current study were also older than the aforementioned study, and this too could influence the frequencies and patterns of behaviors. Finally, all studied behaviors in the second grower stage were positively correlated with flank biting in the finisher stage. Generally, less is known about flank biting and frequencies observed in the current study were higher than earlier reports on Irish pig farms (36). Flank biting was positively correlated between the second grower and finisher stage, as opposed to other damaging behaviors (i.e., ear and tail biting) that did not positively correlate across stages. The reason for this is unknown but it begs the question as to whether other risk factors affect flank biting less or that it has a more stable etiology, compared to ear and tail biting. Flank biting was also correlated with the other behaviors, which could again relate to the restlessness and disturbances remaining within these pens. Different motivational backgrounds exist for tail biting (43) and some pigs possibly specialize in biting behaviors while others include a more general repertoire of “abnormal” behaviors (32). Although the etiology behind tail biting is complex, the development of tail lesions themselves is quite straightforward compared to ear lesions, which have a more complex etiology. Behavior directed toward the ears does not immediately result in ear lesions though the resulting disruptions to the skin in combination with commensal pathogens may lead to ear necrosis (11). More research is needed to understand the relationship between behaviors across stages, ideally for the different types of tail and ear biting that occur from resource competition or agonistic interactions versus ones that occur from a redirection of exploratory behavior (43, 44), and in what situations there are shared or unshared risk factors. Environmental conditions of the barn and consistency across individual pigs should also be investigated. The development of precision livestock farming technologies may aid in this effort, though validation of technologies is needed (26, 44).

The prevalence of pigs with new lesions on transfer to the second grower and finisher stage was highly variable. We previously identified numerous patterns of ear and tail lesion severity in these data suggesting large individual variability in lesion progression (24). We should also acknowledge that lesions might have healed to some extent; however, ear lesions in particular tend to persist throughout the production stages (24). Additionally, in the current study, we focused on pigs with new lesions of any severity compared to no lesions upon transfer to the next production stage. New lesions observed on transfer to the second grower stage occurred in the 2 weeks pigs spent in the first grower stage, while new lesions detected on transfer to the finisher stage occurred during the 4 weeks pigs were in the second grower stage. This suggests that lesions can develop relatively quickly. It is therefore important to have early warning signals that help identify the risk of future lesions and insight into the development of such warning signals.

Frequency of tail biting was identified as the first behavioral indicator that could be used as a warning signal for the development of new tail and ear lesions on arrival to the second grower and finisher stages. Although the frequency of tail biting associated with the development of new lesions is likely to be farm specific, our results suggest that in pens where tail biting is more frequently observed, it is likely that more pigs will develop new tail and ear lesions. This finding is consistent for the development of new tail lesions (i.e., on arrival to the second grower and the finisher stage). In the case of the development of ear lesions, this, however, was only true for the prevalence of ear lesions on arrival to the second grower stage, while the opposite was observed on arrival to the finisher stage where pens with a higher frequency of tail biting had fewer new ear lesions. The importance of the frequency of tail biting for development of new tail lesions is not surprising, while the relationship with ear lesions may be indirect. In pens where tail biting was observed less frequently, ear biting could be used as a secondary warning sign for new lesions. Indeed, in pens with a higher frequency of ear biting there were also more new ear lesions, though this increase in new ear lesions was to a lesser degree than the increase seen in tail lesions associated with tail biting. A higher frequency of ear biting was also associated with fewer new tail lesions on transfer to the next production stage. It is suggested that ear biting occurs more often when tails are docked short (45). In the current study, however, all pigs were tail docked which resulted in largely uniform tail lengths. It may be that in certain pens pigs simply focus on ears over tails or vice versa for unknown reasons, leading to a higher prevalence of the respective lesions in general. This may also be due to the two behaviors possessing different motivational backgrounds. However, the finding that pens with more tail biting in the first grower stage were associated with a higher prevalence of new ear lesions on arrival to the second grower stage, could reflect the role of tail lesions as iceberg indicators, reflecting a wider range of underlying deficiencies, when it comes to pig welfare (3, 46, 47). While we did not measure risk factors in this study per se, pigs were exposed to numerous influential risk factors (i.e., disease, transportation, mixing). Applying the cumulative risk factor bucket framework (48), these factors “filled the pigs” “bucket” to the point where only a small increase in one risk factor would cause the bucket to overflow, resulting in damaging behavior and ultimately the associated visible welfare issues (lesions). The variation between pens suggests that the critical level where an animal’s “bucket” will overflow also varies.

Determining early warning signals to identify pens at high risk of a tail biting outbreak was a promising research development in recent years. However, our results indicate that lesions can develop even at relatively low observed frequencies of ear and tail biting which highlights the considerable difficulty in managing these multifactorial welfare problems. Previous research identified tail biting outbreaks based on the percentage of pigs with severe tail lesions with different definitions or cut-offs in the number of pigs (e.g., 6–24% of pigs in a pen) affected (14, 49). Identifying the lesions themselves is a useful tool but is too late to use as a prevention strategy. Statham et al. (49) found that tail biting increased preceding the outbreaks, though not in all cases. Because ear and tail biting have different motivational backgrounds and etiologies (4, 32, 43), a variety of management strategies are needed. Additionally, the large variability between pens and inconsistency of behavior across production stages indicates that management strategies should potentially be implemented on a pen-level. Such strategies should be implemented as early as possible to prevent or reduce the outbreak of damaging behaviors and their associated lesions (12, 50, 51). Identifying and giving extra attention to high risk periods of the production cycle is also recommended, as our results indicated a potentially sensitive period in the second grower stage on this farm where all four behaviors were strongly correlated to one another. The high variability between pens suggests that some pens (or pigs in these pens) may be more sensitive to challenges than others.

It should be acknowledged that there are more potential early warning signals that were not included in the current study. While predisposing factors for tail biting are well researched, there are no clear or reliable predictors for biting activity further complicated by the inconsistency of pigs expressing the behavior (34, 52). Including other potential warning signals (e.g., tucked tails, environmental conditions) would have been interesting (14, 53, 54), but was out of the scope of the current study. Thus, future research on the development of early warning signals should take this into account. Additionally, the observed thresholds are specific to the context of this study and this should be taken into consideration when interpreting the observed cut-off values. While the cut-off values should not be generalized to universal cut-off values, it can be considered a proof of concept of how thresholds could be developed for specific farms, and may be a good first step for producers to evaluate high risk pens. Our study’s relatively low thresholds provides interesting insight into pig welfare showing that new lesions were associated with relatively low ear and tail biting threshold values which differ depending on production stage. This highlights that thresholds for warning signals may be dynamic and change over time based on the pigs’ age, needs, and husbandry and environmental conditions.

## 5 Conclusion

The results show that behaviors are variable and the relationship between behaviors can change over time. This work emphasizes the intricacies in developing cut-off values for warning signals for damaging behaviors. The findings on this farm emphasize that thresholds are dynamic (i.e., differ per production stage and behavior) and this may relate to the cumulative effect of different risk factors that need to be considered. Furthermore, not only farm specific but also likely pen specific strategies are needed to manage the different behaviors and lesions. This study provided a first proof of concept to aid in the understanding of the development of threshold values in a commercial farm setting.

## Data Availability

The raw data supporting the conclusions of this article will be made available by the authors, without undue reservation.

## Supplementary material

Supplementary Figure 1 Average frequency of **(A)** ear, **(B)** tail, and **(C)** flank biting and **(D)** aggression behaviors performed per 5-min observation in 48 mixed sex groups (*n* = 1,676) during 11 consecutive weeks throughout the first grower (Grower I, 12–13 weeks of age), second grower (Grower II, 14–17 weeks of age), and finisher (18 weeks of age until slaughter) stages in a commercial farm.

Supplementary Figure 2 Prevalence of pigs with new ear lesions for each pen on arrival to the second grower (Grower II, gray dot) and finisher stage (Finisher, red diamond). Arrows indicate whether the prevalence of new lesions increased (upward arrow), remained equal (horizontal arrow), or decreased (downward arrow) from the second grower to the finisher stage.

Supplementary Figure 3 Prevalence of pigs with new tail lesions for each pen on arrival to the second grower (Grower II, gray dot) and finisher stage (Finisher, red diamond). Arrows indicate whether the prevalence of new lesions increased (upward arrow), remained equal (horizontal arrow), or decreased (downward arrow) from the second grower to the finisher stage.
