# Supplementary figures and images for: Observations on the associations between damaging and aggressive behaviors, related lesions, and their implications for the welfare of pigs in the grower-finisher period
Source: Front Vet Sci. 2025 Mar 24;12:1523663. doi: 10.3389/fvets.2025.1523663 (PMC11973387; doi:10.3389/fvets.2025.1523663)

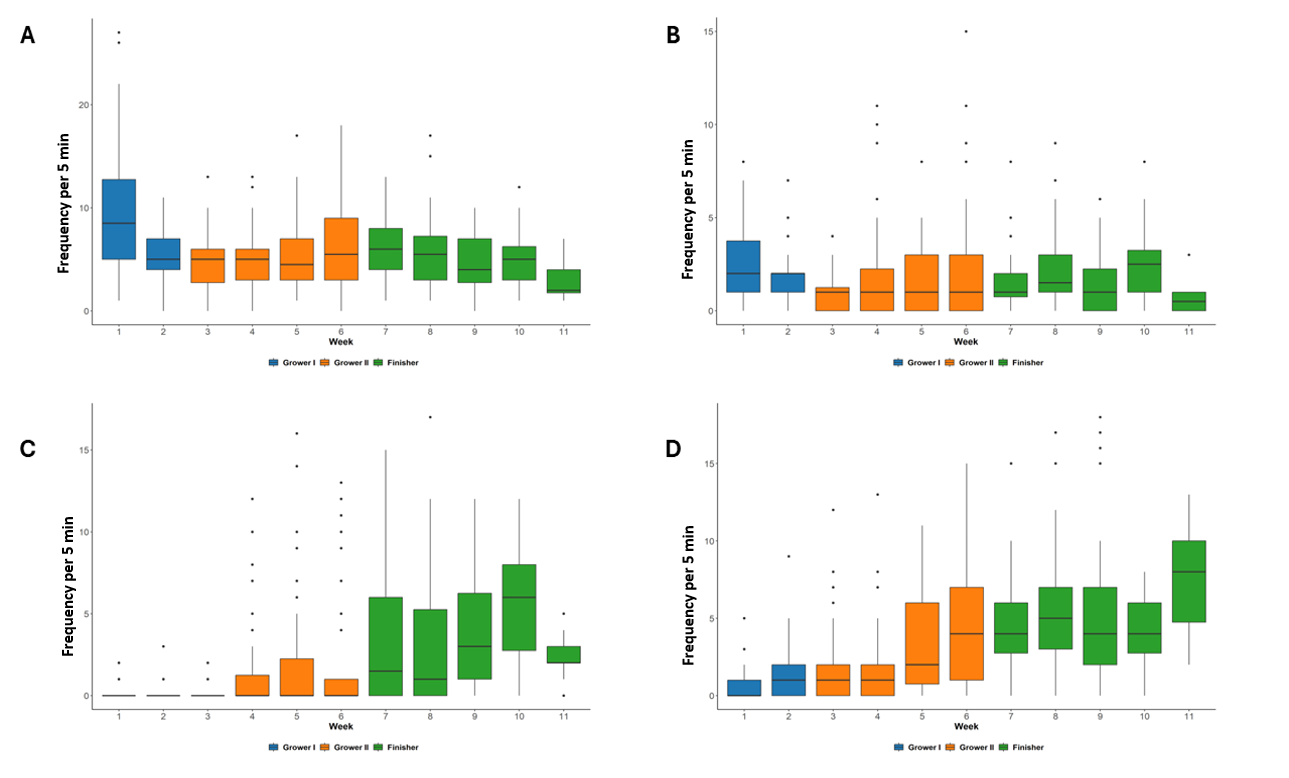

Supplement: Supplementary Figure 1 — Average frequency of (A) ear, (B) tail, and (C) flank biting and (D) aggression behaviors performed per 5-min observation in 48 mixed sex groups (n = 1,676) during 11 consecutive weeks throughout the first grower (Grower I, 12–13 weeks of age), second grower (Grower II, 14–17 weeks of age), and finisher (18 weeks of age until slaughter) stages in a commercial farm. [file Image_1.TIF]

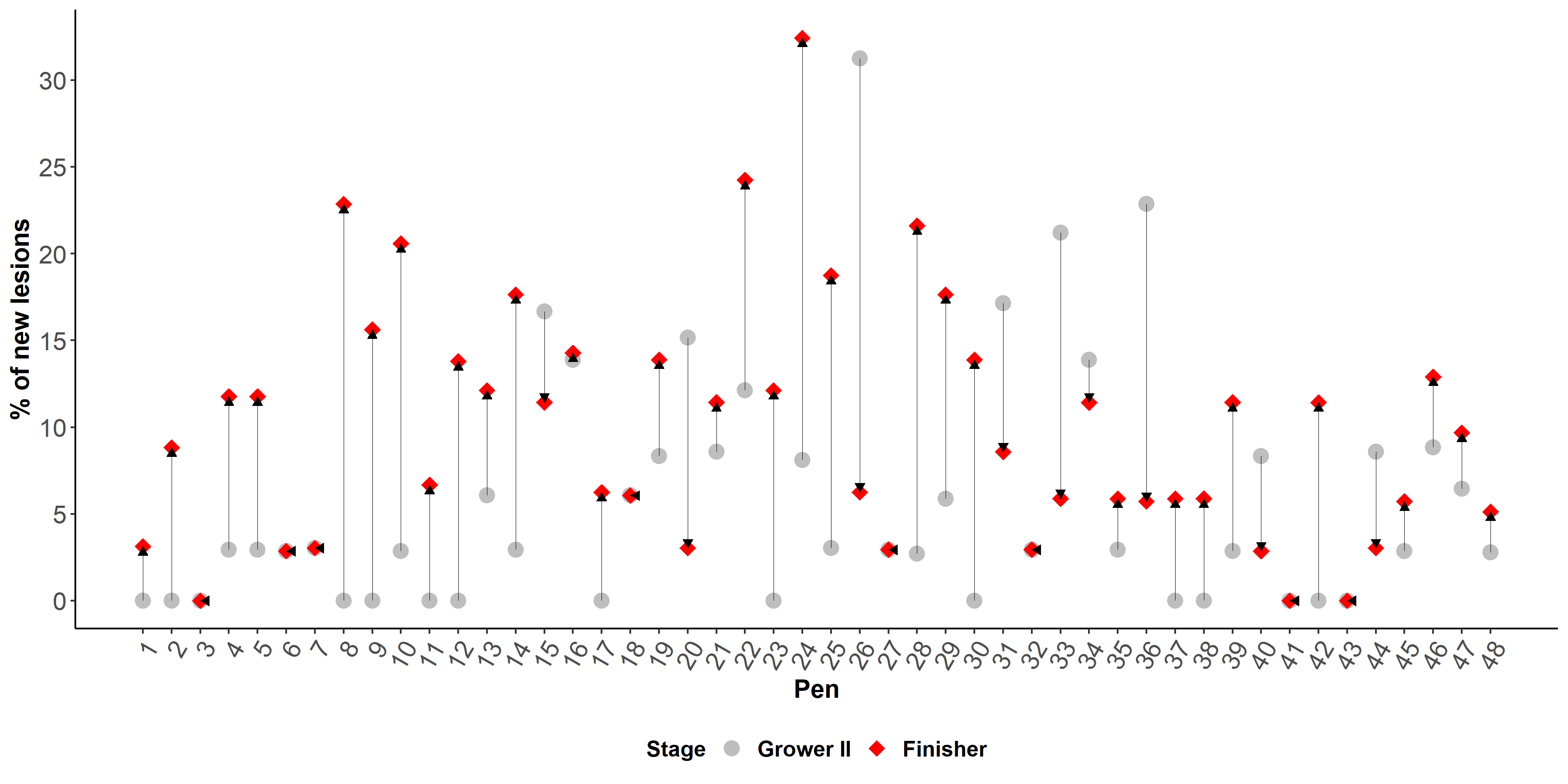

Supplement: Supplementary Figure 2 — Prevalence of pigs with new ear lesions for each pen on arrival to the second grower (Grower II, gray dot) and finisher stage (Finisher, red diamond). Arrows indicate whether the prevalence of new lesions increased (upward arrow), remained equal (horizontal arrow), or decreased (downward arrow) from the second grower to the finisher stage. [file Image_2.TIFF]

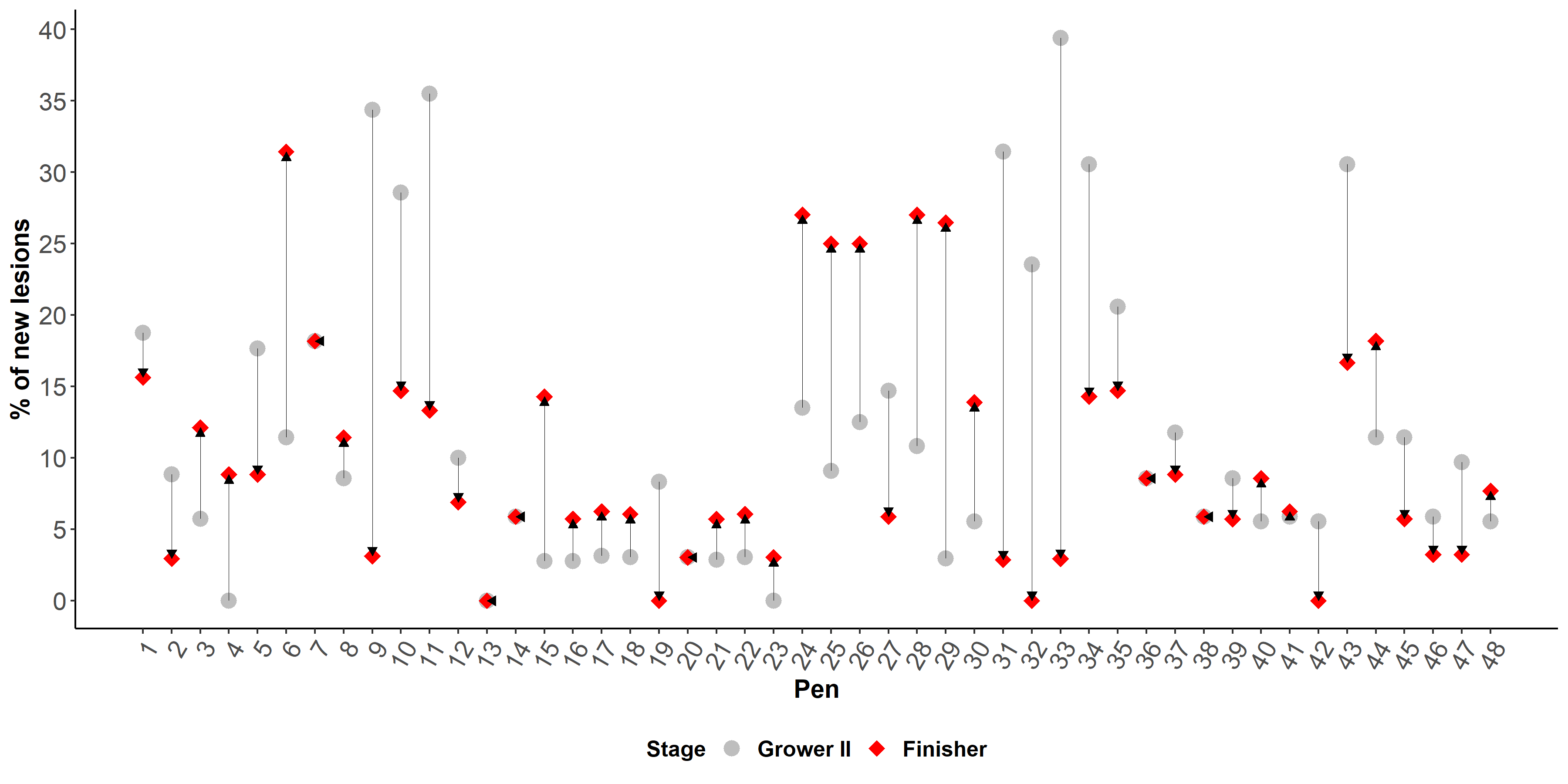

Supplement: Supplementary Figure 3 — Prevalence of pigs with new tail lesions for each pen on arrival to the second grower (Grower II, gray dot) and finisher stage (Finisher, red diamond). Arrows indicate whether the prevalence of new lesions increased (upward arrow), remained equal (horizontal arrow), or decreased (downward arrow) from the second grower to the finisher stage. [file Image_3.TIFF]
